# Incidence and Causes of Early Hospital Readmissions After Living-Donor Renal Transplant at King Abdulaziz Medical City, Riyadh

**DOI:** 10.7759/cureus.40254

**Published:** 2023-06-11

**Authors:** Abdulrahman R Altamimi, Fahad K Alrzouq, Ziad A Aljaafri, Faris Alahmadi, Yousef Alsuwailem, Fares Dendini

**Affiliations:** 1 Department of Transplantation and Hepatobiliary Surgery, Ministry of the National Guard Health Affairs, Riyadh, SAU; 2 Department of Research, King Abdullah International Medical Research Center, Riyadh, SAU; 3 College of Medicine, King Saud Bin Abdulaziz University for Health Sciences, Riyadh, SAU

**Keywords:** rehospitalization, living-donor, complications, early readmission, kidney transplantation

## Abstract

Background: Living-donor organ transplant has a higher long-term survival rate compared to deceased-donor organ transplant, with kidney transplantation being the optimal treatment for most kidney failure patients. However, early hospital readmission within 30 days of discharge can occur due to various factors and can negatively affect long-term outcomes. Effective communication with patients pre-and post-transplant is crucial for a better quality of life and for reducing readmissions. Chronic kidney disease and co-morbid conditions must also be addressed for better long-term outcomes. The incidence and causes of early hospital readmission vary depending on local characteristics and other factors.

Methods: A retrospective cohort study of outcomes in patients who underwent living-donor renal transplantation at King Abdulaziz Medical City (KAMC) between 2015 and 2022. Data were collected by chart review using the BestCare system. The data collected included patients’ demographics, comorbidities, surgery-related data, and the outcome of transplantation. The categorical data were presented using percentages and frequencies, while the numerical data were presented as mean and standard deviation. The Chi-square test was used for inferential statistics to find the association between categorical variables.

Results: Regarding sociodemographic characteristics, the majority of patients were male, aged 19-50 years, and either overweight or had obesity class 1. The incidence of complications, graft failure, and mortality after renal transplant was low, with only a small percentage of patients experiencing these outcomes within one year of transplant. There is no significant association between gender, age, BMI, and the likelihood of readmission after renal transplantation. Patients with comorbidities such as hypertension, diabetes, and coronary artery disease had a higher likelihood of readmission after renal transplantation. The study provides an association between readmission after renal transplantation and various factors such as surgical complications, previous transplant, age at transplant, graft failure, and mortality. Out of the 107 readmitted patients, 2.8% had surgical complications, and 5.6% had a previous transplant, but the association was not statistically significant.

Conclusion: Early hospital readmission within 30 days of discharge can be a concern for patients undergoing renal transplants. While the incidence of complications, graft failure, and mortality after renal transplant was low, patients with comorbidities such as hypertension, diabetes, and coronary artery disease had a higher likelihood of readmission after renal transplantation. Although the association between surgical complications and readmission was not statistically significant, it is important to continue monitoring this factor in future studies. Effective communication with patients pre-and post-transplant can play a crucial role in reducing readmissions and improving long-term outcomes.

## Introduction

Living-donor organ transplant is the best choice when it comes to the survival rate, quality of life, and complications. Previous studies have shown that it has a higher association with long-term survival when compared to deceased-donor organ transplants. Patients with a living-donor organ reach a survival rate of 80% at five years compared to 65% in patients with a deceased-donor organ. Of course, there are special difficulties with getting a donor, the surgery itself, and even after the transplant [1,2].

Kidney transplantation is considered the optimal and definitive treatment for most kidney failure patients. Despite its complexity, it is still preferable for most patients over dialysis [3]. When compared to dialysis, kidney transplantation patients have a better quality of life [4]. However, the first year after the transplant needs close medical follow-up from the transplant center and the nephrologist to ensure the patient has the best outcome from the surgery. Therefore, having effective communication with the patient pre-and post-transplantation will give the patient a better quality of life later and reduce readmissions [4,5]. Usually, these patients are known to have chronic kidney disease; thus, there is a need to address how were the disease progression and co-morbid conditions through a multifaceted approach for the sake of improving the long-term outcomes post-transplant [6].

Early hospital readmission (EHR), defined as readmission within 30 days of discharge, is a measure of the quality of health care [7,8]. It is influenced by several factors, including demographic, socioeconomic, donor, and recipient factors, and could be related to the surgery itself [3,7,9]. A study showed the incidence in two different studies in two different countries. The first was in the USA and it showed EHR ranges from 18 to 47%, and the other one in Brazil showed an incidence of 19.8% [7,10,11]. Mainly, these EHRs were due to infections, surgical and metabolic complications. And those patients who were readmitted early are more likely to experience worse long-term outcomes since the same study showed these patients with EHR had three times the risk of being readmitted in the year after the early admission [7]. Another study done on 1175 patients showed an incidence of EHR of 26.6%, which was mainly caused by infection (67%), surgical complications (14%), and metabolic disturbances (11%). And they concluded that EHR by itself was related to mortality. However, incidence and causes are associated with local characteristics of the population and other factors [9]. Since there are limited studies looking at the EHR, and no studies were conducted locally, we decided to understand the factors associated with EHR at King Abdulaziz Medical City, Riyadh.

## Materials and methods

This study conducted a retrospective cohort study at King Abdulaziz Medical City in Riyadh to investigate living donor renal transplantation outcomes in patients between 2015 and 2022. The study subjects included all patients who underwent living donor renal transplantation during the specified period and had no exclusion criteria. The sampling technique utilized for the study was non-probability consecutive sampling, including all patients who met the inclusion criteria. Data were collected by chart review using the Best-Care system at KAMC, and only the research team members collected the data. The collected data included patient demographics, comorbidities, surgery-related data, and outcomes such as graft failure and mortality within one year of transplantation.

The data collected were managed and analyzed using Microsoft Excel and SPSS statistical software, version 27.0.1 (IBM Corp., Armonk, NY). The categorical data were presented using percentages and frequencies, while the numerical data were presented as mean and standard deviation. The Chi-square test was used for inferential statistics to find the association between categorical variables. The study adhered to ethical considerations by assuring the privacy and confidentiality of the subjects. Informed consent was not required as it was a retrospective study, and no identification data such as medical record numbers, names, and IDs were collected. The access to research data was kept only between the study group members. Overall, this study provided valuable insights into living donor renal transplantation outcomes in the specified study population.

Both descriptive and inferential statistical analysis of the data was carried out. Simple frequencies, and percentages of the sociodemographic characteristics like gender, age, etc. of renal transplant patients were calculated and tabulated. Association between different risk factors, morbidity, mortality, and readmission after Renal Transplant Surgery were calculated with Fischer’s Exact Test. Statistical significance was established at a p-value of 0.05 or less with a 95% confidence interval (CI). All the statistical calculations were performed using the SPSS software, version 27.0.1 (IBM Corp., Armonk, NY).

## Results

Descriptive statistics of sociodemographic features

Table 1 provides sociodemographic features of 557 patients who underwent renal transplants. The patients were classified based on their gender, age, and body mass index (BMI). The gender distribution of the patients shows that 65.4% (364 out of 557) were male and 34.6% (193 out of 557) were female. In terms of age, the majority of the patients were between 19 to 50 years old (51.3%, 286 out of 557), followed by those between 51 to 70 years old (38.2%, 213 out of 557). Only a small percentage of patients were below 18 years (5.7%, 32 out of 557), while 4.7% (26 out of 557) were over 70 years of age. The BMI classification shows that the majority of patients were either overweight (35.7%, 199 out of 557) or had obesity class 1 (25.1%, 140 out of 557). A smaller proportion of patients were classified as normal (23.7%, 132 out of 557) or underweight (5.6%, 31 out of 557). A small percentage of patients were in the obesity class 2 (8.1%, 45 out of 557) or obesity class 3 (1.8%, 10 out of 557) categories.

In summary, this table provides an overview of the sociodemographic features of patients who underwent renal transplants, including their gender, age, and BMI classification. The information presented here can be used to gain insights into the characteristics of patients who undergo renal transplants and to guide clinical decision-making in the future.

**Table 1:** Sociodemographic features of the patients who underwent renal transplants BMI: Body Mass Index

		Frequency (n=557)	Percentage
Gender	Male	364	65.4
	Female	193	34.6
	Total	557	100.0
Age	1-18 Years	32	5.7
	19-50 Years	286	51.3
	51-70 Years	213	38.2
	> 70 Years	26	4.7
	Total	557	100.0
BMI	Underweight	31	5.6
	Normal‎	132	23.7
	Overweight	199	35.7
	Obesity Class 1	140	25.1
	Obesity Class 2	45	8 1
	Obesity Class 3	10	1.8
	Total	557	100.0

Major comorbidities in renal transplant patients

**Figure 1:**
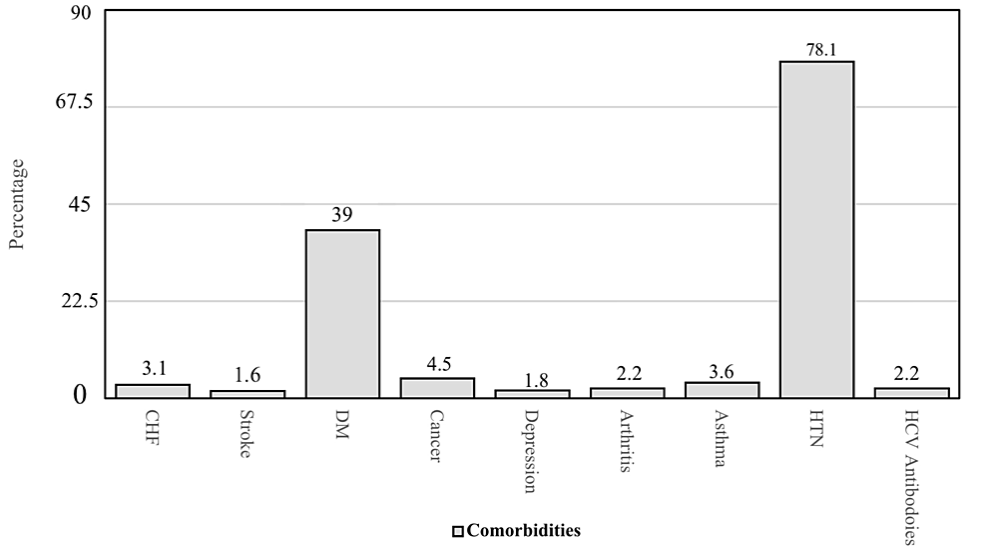
Major comorbidities in renal transplant patients CHF: Congestive Heart Failure, DM: Diabetes Mellitus, HTN: Hypertension, HCV: Hepatitis C Virus

Figure 1 shows the major comorbidities in renal transplant patients. Hypertension is the most common comorbidity in these patients. Diabetes Mellitus is the second most common morbidity in these patients. Other comorbidities are shown in Figure 1. 

Different surgical features of patients who underwent renal transplants, and associated complications rate

Table 2 provides information on the different surgical features of patients who underwent renal transplants, along with the rate of associated complications. The data is based on a sample of 557 patients. The first category is admission 90 days before the transplant, which shows that 24.1% (134 out of 557) of patients were admitted 90 days before their transplant, while the remaining 75.9% (423 out of 557) did not. All of the patients in the sample received a living donor kidney transplant. In terms of age at transplantation, the majority of patients were between 19 to 50 years old (53.9%, 300 out of 557), followed by those between 51 to 70 years old (34.8%, 194 out of 557). Only a small percentage of patients were below 18 years (8.8%, 49 out of 557), while 2.5% (14 out of 557) were over 70 years of age. The incidence of complications was low, with only 1.6% (9 out of 557) of patients experiencing complications after transplant, while the remaining 98.4% (548 out of 557) had no complications.

**Table 2:** Different surgical features of patients who underwent renal transplant and associated complications rate

		Frequency (n=557)	Percentage
Admission 90 Days Before Transplant	Yes	134	24.1
	No	423	75.9
	Total	557	100.0
Living Donor Kidney Transplantation	Yes	557	100.0
Age of Patient at Transplantation	1-18 Years	49	8.8
	19-50 Years	300	53.9
	51-70 Years	194	34.8
	> 70 Years	14	2.5
	Total	557	100.0
Incidence of Complications	Yes	9	1.6
	No	548	98.4
	Total	557	100.0
Previous Transplant	Yes	18	3.2
	No	539	96.8
	Total	557	100.0
Early Hospital Readmission	Yes	107	19.2
	No	450	80.8
	Total	557	100.0
Graft Failure within 1 Year	Yes	2	.4
	No	555	99.6
	Total	557	100.0
Mortality within 1 Year	Yes	3	.5
	No	554	99.5
	Total	557	100.0

Regarding the previous transplant, 3.2% (18 out of 557) of patients had undergone a previous transplant, while 96.8% (539 out of 557) had not. A total of 19.2% (107 out of 557) of patients required early hospital readmission after their transplant, while the remaining 80.8% (450 out of 557) did not.

The graft failure rate within one year after transplant was low, with only 0.4% (two out of 557) of patients experiencing graft failure, while the remaining 99.6% (555 out of 557) did not experience any graft failure. The mortality rate within one year after transplant was also low, with only 0.5% (three out of 557) of patients experiencing mortality, while the remaining 99.5% (554 out of 557) did not experience any mortality.

Major indications for renal transplant surgery in renal disease patients

**Figure 2:**
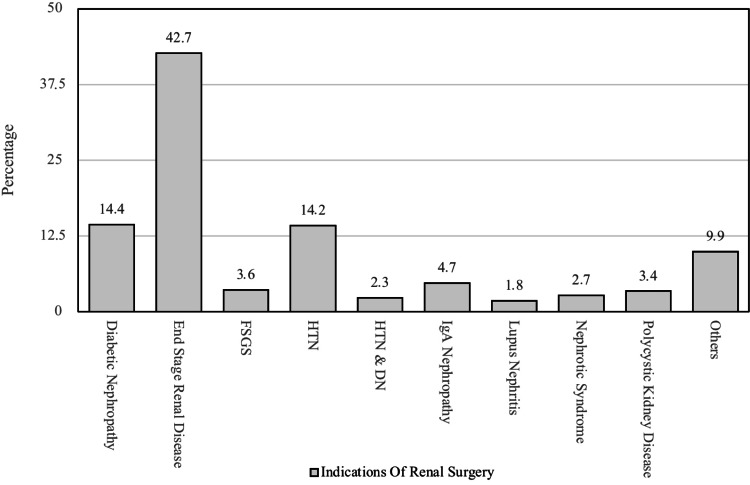
Major indication for renal transplant surgery in renal disease patients FSGS: Focal Segmental Glomerulosclerosis, HTN: Hypertension, HTN & DN: Hypertension & Diabetic Nephropathy

Figure 2 shows the major indications for renal transplant surgery in patients. End Stage Renal Disease (ESRD) is the most common indication in these patients. Diabetic nephropathy is the second most common morbidity in these patients. Hypertension is the third most common indication for transplant in these patients. Other indications are shown in Figure 2.

Major types of complications in patients after renal transplant surgery

**Figure 3:**
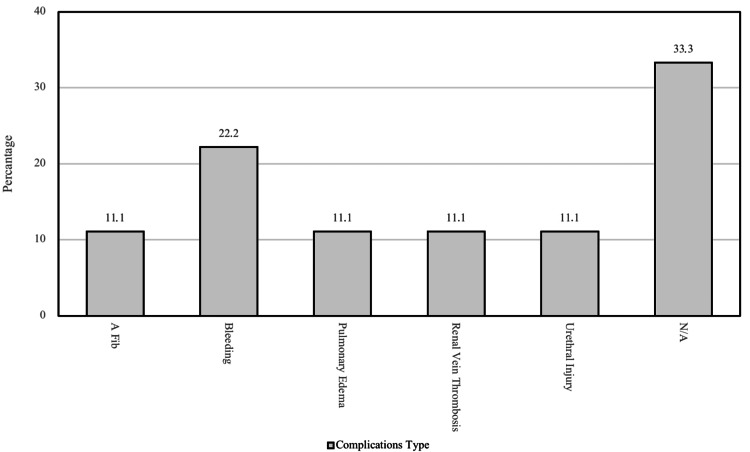
Major types of complications in patients after renal transplant surgery A Fib: Atrial Fibrillation, N/A: Not Applicable

Figure 3 shows the major complications of renal transplant surgery in patients. Bleeding is the most common complication in these patients. Other complications are shown in Figure 3.

Major reasons for readmission in patients after renal transplant surgery

**Figure 4:**
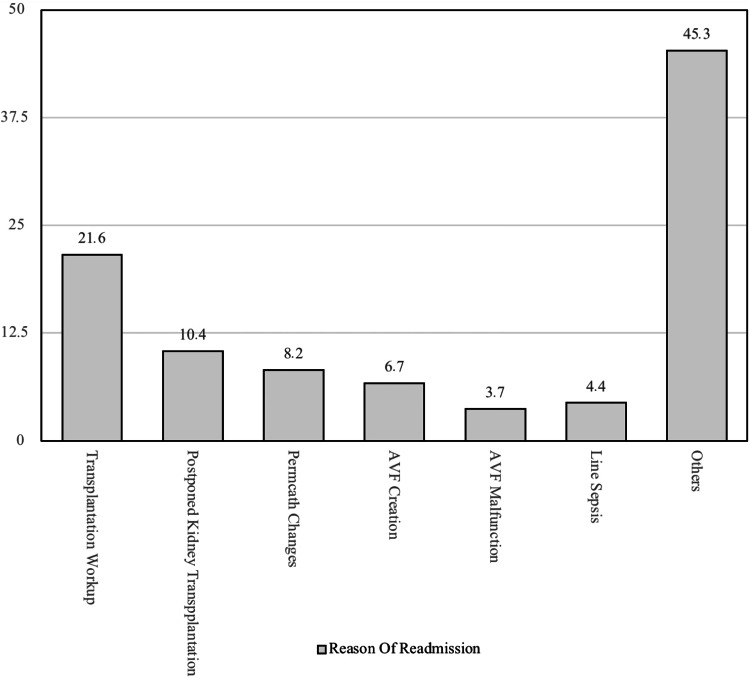
Major reasons for readmission in patients after renal transplant surgery AVF: Arteriovenous Fistula

Figure 4shows the major reasons for readmission after renal transplant surgery in patients. Out of the total, 21.6 % of patients were readmitted due to transplantation workup followed by 10.4 % due to postponed kidney transplantation, followed by permcath changes. Other reasons are shown in Figure 4. 

Factors responsible for early readmission in renal transplants patients

Table 3 presents the association between readmission after renal transplantation and different sociodemographic features of the patients. The table shows the number and percentage of patients who were readmitted after renal transplantation and those who were not, categorized by gender, age, and BMI. In terms of gender, out of the 364 male patients, 67 (62.6%) were readmitted, while out of the 193 female patients, 40 (37.4%) were readmitted. However, the statistical analysis (significant value = 0.500) suggests that there is no significant association between gender and readmission after renal transplantation. Regarding age, the highest proportion of readmission was found in patients aged 19-50 years (46.7%), followed by those aged 51-70 years (42.1%), 1-18 years (6.5%), and >70 years (4.7%). However, the statistical analysis (significant value = 0.710) suggests that there is no significant association between age and readmission after renal transplantation. In terms of BMI, the highest proportion of readmission was found in patients classified as overweight (33.6%), followed by those in obesity class 1 (28.0%), normal weight (23.40%), obesity class 2 (10.3%), and underweight (4.7%). However, the statistical analysis (significant value = 0.553) suggests that there is no significant association between BMI and readmission after renal transplantation. Overall, the results suggest that there is no significant association between the gender, age, and BMI of the patients and their likelihood of readmission after renal transplantation.

**Table 3:** Association between readmission after renal transplantation with different sociodemographic features BMI: Body Mass Index, Sig. Value: Significant Value

		Readmission After Renal Transplantation		Sig. Value
		Yes	No	
Sociodemographic				
Gender	M	67(62.6%)	297(66.0%)	0.500
	F	40(37.4%)	153(34.0%)	
	Total	107(100.0%)	450(100.0%)	
Age	1-18 Years	7(6.5%)	25(5.6%)	0.710
	19-50 Years	50(46.7%)	236(52.4%)	
	51-70 Years	45(42.1%)	168(37.3%)	
	> 70 Years	5(4.7%)	21(4.7%)	
	Total	107(100.0%)	450(100.0%)	
BMI	Underweight	5(4.7%)	26(5.8%)	0.553
	Normal‎	25(23.4%)	107(23.8%)	
	Overweight	36(33.6%)	163(36.2%)	
	Obesity Class 1	30(28.0%)	110(24.4%)	
	Obesity Class 2	11(10.3%)	34(7.6%)	
	Obesity Class 3	0(0.0%)	10(2.2%)	
	Total	107(100.0%)	450(100.0%)	

Table 4 shows the association between readmission after renal transplantation and different comorbidities. The table presents the number and percentage of patients who were readmitted and not readmitted after renal transplantation based on the presence or absence of comorbidities. For congestive heart failure (CHF), four (3.7%) patients who were readmitted had CHF compared to 13 (2.9%) patients who were not readmitted. This difference was not statistically significant (p=0.753). Similarly, for stroke, three (2.8%) patients who were readmitted had a history of stroke, compared to six (1.3%) patients who were not readmitted. However, this difference was also not statistically significant (p=0.384).

**Table 4:** Association between Readmission after Renal Transplantation with different Sig. Value: Significant Value, CHF: Congestive Heart Failure, DM, Diabetes Mellitus, HTN: Hypertension, HCV: Hepatitis C Virus

		Readmission After Renal Transplantation		Sig. Value
		Yes	No	
Comorbidities				
CHF	Yes	4(3.7%)	13(2.9%)	0.753
	No	103(96.3%)	437(97.1%)	
	Total	107(100.0%)	450(100.0%)	
Stroke	Yes	3(2.8%)	6(1.3%)	0.384
	No	104(97.2%)	444(98.7%)	
	Total	107(100.0%)	450(100.0%)	
DM	Yes	47(43.9%)	170(37.8%)	0.270
	No	60(56.1%)	280(62.2%)	
	Total	107(100.0%)	450(100.0%)	
Cancer	Yes	6(5.6%)	19(4.2%)	0.602
	No	101(94.4%)	431(95.8%)	
	Total	107(100.0%)	450(100.0%)	
Depression	Yes	4(3.7%)	6(1.3%)	0.105
	No	103(96.3%)	444(98.7%)	
	Total	107(100.0%)	450(100.0%)	
Arthritis	Yes	4(3.7%)	8(1.8%)	0.258
	No	103(96.3%)	442(98.2%)	
	Total	107(100.0%)	450(100.0%)	
Asthma	Yes	4(3.7%)	16(3.6%)	1.000
	No	103(96.3%)	434(96.4%)	
	Total	107(100.0%)	450(100.0%)	
HTN	Yes	79(73.8%)	356(79.1%)	0.243
	No	28(26.2%)	94(20.9%)	
	Total	107(100.0%)	450(100.0%)	
HCV Antibodies	Yes	3(2.8%)	9(2.0%)	0.709
	No	104(97.2%)	441(98.0%)	
	Total	107(100.0%)	450(100.0%)	
Duration of Dialysis	< 1 year	30(34.5%)	114(33.3%)	0.127
	1-5 Years	51(58.6%)	185(54.1%)	
	6-10 Years	4(4.6%)	40(11.7%)	
	> 10 Years	2(2.3%)	3(0.9%)	
	Total	87(100.0%)	342(100.0%)	

For diabetes mellitus (DM), 47 (43.9%) patients who were readmitted had DM, compared to 170 (37.8%) patients who were not readmitted. However, this difference was not statistically significant (p=0.270). For cancer, six (5.6%) patients who were readmitted had cancer, compared to 19 (4.2%) patients who were not readmitted. This difference was also not statistically significant (p=0.602). For depression, four (3.7%) patients who were readmitted had depression, compared to six (1.3%) patients who were not readmitted. This difference was not statistically significant (p=0.105). Similarly, for arthritis, four (3.7%) patients who were readmitted had arthritis, compared to eight (1.8%) patients who were not readmitted. However, this difference was not statistically significant (p=0.258). For asthma, four (3.7%) patients who were readmitted had asthma, compared to 16 (3.6%) patients who were not readmitted. This difference was not statistically significant (p=1.000). For hypertension (HTN), 79 (73.8%) patients who were readmitted had HTN, compared to 356 (79.1%) patients who were not readmitted. However, this difference was not statistically significant (p=0.243). For HVC antibodies, three (2.8%) patients who were readmitted had HCV antibodies, compared to nine (2.0%) patients who were not readmitted. This difference was not statistically significant (p=0.709). Finally, for the duration of dialysis, patients who had undergone dialysis for 6-10 years had the highest proportion of readmissions (4.6%), followed by those who had undergone dialysis for less than one year (34.5%), and those who had undergone dialysis for 1-5 years (58.6%). The difference in readmission rates based on the duration of dialysis was not statistically significant (p=0.127).

Table 5 shows the association between readmission after renal transplantation and different surgical factors, graft failure, and mortality. The table shows the number and percentage of patients who were readmitted and those who were not readmitted for each factor. The significance value (Sig. Value) is also provided to indicate if the association is statistically significant or not. Regarding complications of transplant surgery, where three out of 107 patients (2.8%) who were readmitted had complications, while only six out of 450 patients (1.3%) who were not readmitted had complications. However, the association was not statistically significant (p=0.384). Regarding previous transplants, where six out of 107 patients (5.6%) who were readmitted had a previous transplant, while only 12 out of 450 patients (2.7%) who were not readmitted had a previous transplant. The association was not statistically significant (p=0.131). Regarding the age at transplant, where the patients were divided into four age groups. The highest percentage of readmission was observed in the age group of 19-50 years (51.4%), followed by the age group of 51-70 years (37.4%), and the age group of 1-18 years (9.3%). Only two patients in the age group of >70 years were readmitted. However, the association was not statistically significant (p=0.894). Regarding graft failure within one year, two out of 107 patients (1.9%) who were readmitted experienced graft failure within one year, while none of the patients who were not readmitted had graft failure. The association was statistically significant (p=0.037*). Regarding mortality within one year, two out of 107 patients (1.9%) who were readmitted died within one year, while only one out of 450 patients (0.2%) who were not readmitted died within one year. However, the association was not statistically significant (p=0.096).

**Table 5:** Association between readmission after renal transplantation with different surgical Factors, graft failure, and mortality Sig. Value: Significant Value

			Readmission After Renal Transplantation		Sig. Value
			Yes	No	
Surgical Factors					
Complications of Transplant Surgery	Yes	3(2.8%)		6(1.3%)	0.384
	No	104(97.2%)		444(98.7%)	
	Total	107(100.0%)		450(100.0%)	
Previous Transplant	Yes	6(5.6%)		12(2.7%)	0.131
	No	101(94.4%)		438(97.3%)	
	Total	107(100.0%)		450(100.0%)	
Age at Transplant	1-18 Years	10(9.3%		39(8.7%)	0.894
	19-50 Years	55(51.4%		245(54.4%)	
	51-70 Years	40(37.4%		154(34.2%)	
	> 70 Years	2(1.9%		12(2.7%)	
	Total	107(100.0%		450(100.0%)	
Graft Failure within 1 Year	Yes	2(1.9%)		0(0.0%)	0.037*
	No	105(98.1%)		450(100.0%)	
	Total	107(100.0%)		450(100.0%)	
Mortality within 1 year	Yes	2(1.9%)		1(0.2%)	0.096
	No	105(98.1%)		449(99.8%)	
	Total	107(100.0%)		450(100.0%)	

## Discussion

Our study reveals that the majority of patients who underwent renal transplants were male and between the ages of 19 to 50 years old. This finding is consistent with previous studies showing a higher incidence of renal disease among males and younger individuals [12]. The higher incidence of renal disease among males may be due to a combination of genetic and environmental factors, such as higher rates of hypertension and diabetes in men.

Additionally, the BMI classification of patients who underwent renal transplants suggests that overweight and obesity are common among this patient population. This finding is also consistent with previous studies showing a high prevalence of overweight and obesity in patients with renal disease, which can contribute to the development and progression of renal disease [13].

The incidence of complications, graft failure, and mortality rates in renal transplant patients in this sample were low. Only 107 (19.2%) of patients required readmission within 30 days after transplant. These findings are consistent with previous studies that have reported favorable outcomes in renal transplant recipients [14]. It is due to the fact that a functioning kidney transplant can provide improved quality of life and better long-term survival compared to long-term dialysis. Kidney transplantation can restore normal kidney function, allowing for improved control of blood pressure and electrolyte balance, as well as improved cardiovascular health.

It is important to note that the sample only included patients who received a living donor kidney transplant, which may have contributed to the low rates of complications and mortality observed. Living donor kidney transplants have been associated with better outcomes compared to deceased donor kidney transplants [15]. However, it is also important to consider that the sample size was relatively small, and further studies with larger sample sizes are needed to confirm these findings.

The age distribution of patients in this sample is consistent with previous studies that have reported that the majority of renal transplant recipients are between 19 to 50 years old [16]. It is important to note that the age of the donor and recipient may also impact the outcomes of the transplant, and future studies should consider the age of both the donor and recipient when examining outcomes.

Regarding the association between sociodemographic factors and readmission after renal transplantation, the results suggest that there is no significant association between the gender, age, and BMI of the patients and their likelihood of readmission after renal transplantation. These findings are consistent with some previous studies, which also found no significant association between gender and readmission after renal transplantation [17]. It is because the factors that influence readmission rates are more related to medical complications and other individual patient characteristics rather than gender. However, some other studies have reported conflicting results regarding the association between gender and readmission after renal transplantation [18]. Similarly, previous studies have also reported conflicting results regarding the association between age and readmission after renal transplantation [19]. Some studies have found a positive association between age and readmission after renal transplantation, while others have found no association.

In the case of the association between different comorbidities and readmission after renal transplantation, the results suggest that there is no significant association between most of the comorbidities and readmission after renal transplantation. However, patients who had undergone dialysis for less than one year had a higher proportion of readmissions than those who had undergone dialysis for longer durations. This finding is consistent with previous studies, which have reported that patients who undergo transplantation after a shorter duration of dialysis have a higher risk of readmission [20]. It is because they may not have had adequate time to fully recover from the effects of their kidney disease and related comorbidities. These patients may have more severe diseases, more comorbidities, and may require more complex medical management after transplantation, which can increase the risk of readmission.

Further, the association between surgical factors, graft failure, and mortality and readmission after renal transplantation, the results suggest that there is no significant association between complications of transplant surgery, previous transplant, and age at transplant and readmission after renal transplantation. However, patients who had experienced graft failure had a significantly higher proportion of readmissions than those who did not experience graft failure. This finding is consistent with previous studies, which have reported that graft failure is a significant risk factor for readmission after renal transplantation [21]. It is because graft failure can lead to a recurrence of end-stage renal disease and the need for further dialysis or repeat transplantation. Additionally, patients who died after transplantation had a significantly higher proportion of readmissions than those who did not die. Mortality is a significant risk factor for readmission after renal transplantation patients. It is because patients who die following transplantation are no longer able to benefit from the improved renal function provided by the transplanted kidney. In addition, mortality may be associated with medical complications or comorbidities that can increase the risk of readmission for surviving patients.

Since the data of this study was collected from one center, it limits the generalizability of the discoveries. And to achieve accurate results, the subject requires more investigation and research at the local and international levels with multiple centers and a bigger sample size.

## Conclusions

Early hospital readmission within 30 days of discharge can be a concern for patients undergoing a renal transplant. While the incidence of complications, graft failure, and mortality after renal transplant was low, patients with comorbidities such as hypertension, diabetes, and coronary artery disease had a higher likelihood of readmission after renal transplantation. Although the association between surgical complications and readmission was not statistically significant, it is important to continue monitoring this factor in future studies. Effective communication with patients pre-and post-transplant can play a crucial role in reducing readmissions and improving long-term outcomes.
